# Moxibustion therapy on myofascial pain syndrome: An evidence-based clinical practice guideline

**DOI:** 10.1097/MD.0000000000022342

**Published:** 2020-11-13

**Authors:** Zenan Wu, Guixing Xu, Jun Xiong, Zhengyun Zuo, Xinyu Yu, Qiongshan Xie

**Affiliations:** a Jiangxi University of Traditional Chinese Medicine, Nanchang; b Chengdu University of Traditional Chinese Medicine, Nanchang, Chengdu; c The Affiliated Hospital of Jiangxi University of Traditional Chinese Medicine, Nanchang, China.

**Keywords:** moxibustion clinical practice guideline, myofascial pain syndrome

## Abstract

**Background:**

Myofascial pain syndrome (MPS) is a chronic systemic pain disorder. Among the common treatments, moxibustion has an irreplaceable therapeutic effect and is an effective Traditional Chinese Medicine therapy for MPS. However, the lack of clinical practice guidelines (CPGs) has prompted the publication of guidelines on the use of moxibustion in the treatment of MPS.

**Methods:**

The clinical practice guideline will base on the Institute of Medicine, the World Health Organization guideline handbook, the Grade of Recommendations Assessment, Development, and Evaluation the Appraisal of Guidelines for Research & Evaluation II, Reporting Items for practice, Guideline in Healthcare and recommendations thereof will be made on the basis of systematic reviews. We will establish a guidelines development team that will draft clinical questions in the form of population, intervention, comparison, results and conduct a literature search and quality of evidence assessment. The experts will make recommendations after 2 or 3 rounds of Delphi investigations. We will carefully consider the patient's values and preferences and conduct a peer review.

**Ethics and dissemination:**

The guidelines will not contain any personal data and will not prejudice individual rights, so no ethical approval will be required. The guidelines will be subject to rigorous peer review and may be published in a journal or circulated at relevant conferences.

**Results:**

The guidelines will be published in relevant peer-reviewed journals.

**Conclusion:**

This guideline will make it easier for clinicians to treat MPs in the clinical setting and improve the effectiveness of treatment for MPS.

**Study registration:**

The study is registered with the International Practice Guideline Registry Platform (IPGRP): IPGRP-2020CN030

## 1 Introduction

### 1.1 Description of the condition

Myofascial pain syndrome (MPS) is refers to the muscle and fascia of aseptic inflammation, which is an acute attack of myofascial inflammation due to the body being exposed to external factors, such as wind chill, fatigue, trauma, or inadequate sleep.^[1]^ The acute or chronic injury of the muscles, ligaments and capsule of shoulder, neck and waist is the basic cause of this disease; Clinically, chronic muscle pain, tenderness, and weakness are the main manifestations.^[2]^ In some case, those conditions severely affect the quality of people's life.

MPS tends to occur in people between the ages of 30 and 50. The disease is common in clinical practice.^[3]^ According to the relevant investigation, the incidence of MPS is as high as 30% to 93.0%,^[4]^ the number of people suffering from the disease is increasing in China, and the incidence rate in other countries is also increasing year by year.

The treatment principles of MPS include eliminating the cause, improving local blood circulation and supply, anti-inflammatory and analgesia, eliminating tenderness, and exercise. Many drugs have been used in the treatment of MPS, which include muscle relaxants, benzodiazepines, nonsteroidal anti-inflammatory drugs, antidepressants, and topical analgesics. However, the side effects of these drugs are relatively large, leading to other related problems in clinical practice, and their application scope is increasingly limited.^[5]^ There is an urgent need for a new alternative therapy to supplement it.

### 1.2 Description of the intervention

Moxibustion has a significant effect on analgesia of MPS, with less trauma, less risk, and less adverse reactions.^[6]^ Nowadays, the indications for moxibustion are gradually expanded because of its function and practicality, The heat generated by moxibustion can effectively play the role of replenishing Yang and dispelling cold, promoting blood circulation and removing blood stasis, and warm the meridians and collaterals.^[7]^ It has been found in modern studies that the warm effect and light radiation produced by moxibustion are one of the most important factors in the effective treatment of MPS. The heat of combustion can promote blood circulation, relax blood vessels, and regulate the body's immune system. The far infrared ray produced by moxibustion can directly act on the superficial part of the human body and diffuse the heat by conduction. The near infrared ray energy is strong, can penetrate into the deep tissue directly, provides the necessary energy for the body cell activity.^[8]^

### 1.3 Description of the objectives

This study aims to develop a protocol for the evidence-based clinical practice guideline (CPG) about moxibustion treatment for MPS. The CPG will set a standard of moxibustion treatment for MPS.

## 2 Methods

### 2.1 Principle

The CPG will be stickily performed according to the World Health Organization guidelines,^[9]^ Appraisal of Guidelines for Research & Evaluation II instrument,^[10]^ Grade of Recommendations Assessment, Development, and Evaluation (GRADE) system,^[11]^ and Reporting Items for practice, Guideline in Healthcare checklist.^[12]^

### 2.2 Participating institutions, end-users, and target population

The CPG was launched at the Jiangxi University of traditional Chinese medicine (TCM) and the Affiliated Hospital of Jiangxi University of TCM. Two organizations which share expertise and perspectives will participate in the study. “Moxibustion therapy for MPS: guidelines for evidence-based clinical practice” will be the title of the guidelines which target end-users are acupuncturists, physicians, and journal editors. And target population include the patients with MPS, and the person who can be treated with moxibustion comprise the target population. The guideline contents will include the selection of moxibustion methods, the safety and effectiveness of moxibustion for MPS. And the contents are determined after the preliminary development of the guideline development group and the approval of the guideline steering group.

### 2.3 Guideline working group

The Guideline working group set up in March 2020, made up of the Guideline Steering Group, the Guideline Development Group, and the Guideline Secretary Group. Twenty professional members, from different regions and different genders, which fully ensures fair representation by gender and region, will be included in the guidelines development team, as follow: 10 acupuncturists (especially professional MPS), 2 medical clinicians, 1 General Surgeon, 2 TCM physicians, 2 physiotherapists, 1 nurse, 1 health economist physician, and an editor. The tasks of the Guideline Development Group was as follows:

(1) confirm the scope of the guideline, draft the population, intervention, comparison, outcomes (PICOs);
(2) assess the quality of the evidence;
(3) prepare preliminary recommendations;
(4) make the draft guideline; and
(5) publish and popularize the guideline.

The Guideline Steering Group will consist of 6 members, including 2 acupuncturists, 1 TCM physician, 1 physiotherapist, 1 evidence-based medicine expert, and a health economist physician. The tasks of the Guideline Steering Group as follows:

(1) ratify the PICOs;
(2) direct the literature search and systematic reviews;
(3) check the grade of the evidence;
(4) use the improved Delphi approach to draft the final proposal;
(5) confirm the announcement of the guideline.

The Guideline Secretary Group will consist of 5 members, including 2 evidence-based medicine experts, 2 acupuncturists, and a statistician. The tasks of the Guideline Secretary Group were as follows:

(1) complete a literature search and complete systematic reviews;
(2) Record the patients’ views and preferences.

### 2.4 Declaration of interests and funding support

All members of the working group on the guidelines will be required to complete a conflict of interest declaration form to prevent potential conflicts of interest. This work is supported by a grant from: Jiangxi university of TCM 1050 youth talent project (Grant number: 5141900101), Project of Key Research and Development Project of Jiangxi (20161BBG70109), Jiangxi outstanding young talents funding scheme, China (Grant number: 20171BCB23093), Jiangxi Youth Science Foundation Key Project (Grant number: 20192ACB21007), Jiangxi young Jinggang Scholars Award Program, China (Grant number: Ganjiao Dengzi [2018]no. 82).

### 2.5 Identifying questions and selecting outcomes

The PICOs will be determined after the scope of the guidelines developed by the Guideline Development Group and confirmed by the Guideline Steering Group. The outcomes will be selected and categorized according to their importance by Guideline Development Group. The scores of the results are divided into 3 categories from 1 to 9, roughly as follows: 7 to 9 will be classified as classic, 4 to 6 as important, and 1 to 3 as unimportant.^[11]^ We will formulate problems based on the principles of PICOs, such as:

Can moxibustion be applied to patients with MPS?

P: all patients with MPS
I: patients who received moxibustion therapy
C: patients who not received moxibustion therapy
O: MPS quality of life questionnaire; the treatment efficacy rate; the Pain relief rate; VAS: patients fill in scores on a form according to the severity of their symptoms, from 0 to 10 in order from mild to severe, 0 represents no symptoms, and 10 represents the most serious symptoms; adverse effects.

Which treatment effect is better, moxibustion treatment group or non-moxibustion treatment group?

P: All the patients had MPS
I: Patients treated with moxibustion alone
C: Patients treated with any moxibustion
O: Effective rate of treatment.

Which of the following is better: Moxibustion treatment and conventional drug treatment?

P: All the patients had MPS
I: Patients treated with moxibustion alone
C: Patients treated with conventional drug
O: Effective rate of treatment.

Which treatment effect is best: Moxibustion treatment effect and acupuncture treatment effect?

P: All the patients had MPS
I: Patients treated with moxibustion alone
C: Patients treated with acupuncture alone
O: Effective rate of treatment.

Which treatment effect is best, the conventional treatment acupoint or the special treatment acupoint?

P: All the patients had MPS
I: Patients treated with conventional acupuncture points
C: Patients treated with special acupuncture points
O: Effective rate of treatment.

Is the effect of simple moxibustion therapy and moxibustion combined with other therapies very different?

P: All the patients had MPS
I: Patients treated with moxibustion alone
C: Patients treated with moxibustion combined with other methods
O: Effective rate of treatment.

Moxibustion in which stage of the disease intervention treatment is the best?

P: All the patients had MPS
I: Patients treated with moxibustion in the early and middle stages of the disease
C: Patients treated with moxibustion in the middle and late stages of the disease
O: Effective rate of treatment.

What is the relationship between the amount of moxibustion and the effective rate?

P: All the patients had MPS
I: Patients with high moxibustion quantity
C: Patients treated with moxibustion in the middle and late stages of the disease
O: Effective rate of treatment.

### 2.6 Evidence retrieval and synthesis

#### 2.6.1 Databases

We are going to use systematic electronic search, including PubMed, MEDLINE, Cochrane library, SinoMed, China National Knowledge Infrastructure, WangFang Database, and Chinese Scientific Journal Database from the inception dates to March 31, 2020, and conducted classification and data extraction

#### 2.6.2 Search terms

In order to ensure the comprehensiveness and accuracy of the literature retrieval, we will combine the Suggestions of evidence-based medicine experts with the actual situation in the literature retrieval process to formulate the retrieval strategy, and make corresponding records to find the most appropriate retrieval strategy. The free words and MeSH terms will be combination used with “moxibustion” and “Myofascial Pain Syndrome.” We used the search terms (meta-analysis or randomized controlled trial or retrospective study or practice guideline or observational study or outcome research or clinical article or systematic review) AND (myofascial pain syndrome or Myofascial Pain Syndromes) AND (moxibustion or indirect moxibustion or suspended moxibustion, or direct moxibustion or mild moxibustion or heat-sensitive moxibustion). The language of publication will be unrestricted.

#### 2.6.3 Pilot search

The authors of review conducted pre-testing in order to improve the consistency of literature selection criteria and reduce unnecessary problems in the literature selection process. We obtained the results through article screening and summarized the potential causes of inconsistencies, thus greatly improving the understanding of all authors on inclusion criteria and exclusion criteria.

#### 2.6.4 Literature selection

We divided all relevant literature into 2 parts. The first part includes systematic review, meta-analysis, and network meta-analysis. PRISMA^[13]^ will be used to evaluate the quality of these studies. The second part is the original study, which will be evaluated by CONSORT^[14]^ and risk of bias.

#### 2.6.5 Evidence syntheses

A systematic review published within 3 years will be used directly according PRISMA guidelines. At the same time, Members of the guideline development team will be responsible for assessing the quality of the literature and producing a simple report for discussion at the meeting.

#### 2.6.6 Evidence assessment

The Cochrane risk of bias tool will be used to assess the quality of the included subjects in the following 6 areas:

(1) random sequence production;
(2) distribution of concealment;
(3) blind method (patients, medical staff, result evaluation, and data analysis); Data integrity (follow-up rate and important indicators);
(4) selective reporting (hiding negative results or false positive results);
(5) Other sources of bias (such as unbalanced baseline, suspected fraud, etc).

Two researchers will be judged based on the above evaluation indicators. A “yes” indicates a low risk of bias, a “no” indicates a high risk of bias, and a “unclear” indicates an uncertain risk of bias. A summary of findings table will be generated and included in the final report.^[11,15]^

### 2.7 Patients’ values and preferences

We consulted MPS patients to investigate their value and preference for moxibustion. Based on the findings, the guidelines steering group and the guidelines development group make recommendations that take fully into account the values and preferences of patients. In this study, we will assess the reliability and acceptability of the questionnaire. At the same time, before accepting the survey, the patients need to sign the informed consent, and receive related knowledge training to complete the survey more scientifically.

### 2.8 Developing recommendations

After the evidence is evaluated by the GRADE, the quality of the evidence, the weights of strengths and weaknesses, and the patient's values and preferences will be carefully considered by The Guideline Development Group to develop preliminary recommendations. This proposal will be reviewed by 2 to 3 rounds of Delphi process,^[16]^ which will then be submitted to the Guideline Steering Group for approval. We will use the GRADE Grid instrument^[17]^ to review each recommendation one by one to group it into 1 of 5 options, including “strong recommendation,” “weak recommendation,” “unclear recommendation,” “weak no recommendation,” and “strong no recommendation.” The aim is to reach a better consensus. If 75% of the experts agree on an option, there is consensus on the recommendation. Otherwise, the project goes to the next Delphi process to discuss the disputed project again. Finally all approved recommendations are submitted to the Guideline Steering Group to guide the team to the next step of the approval process.

### 2.9 Peer review

The peer review of the guidelines will be conducted by external experts, and the review process will be carefully documented by the Guideline Development Group, which will then discuss and respond to the recommendations of outside experts.

### 2.10 Publishing of the guideline

The recommended format for the essential RIGHT working group will be used for the guidelines and is expected to be published in both English and Chinese in the relevant Chinese and English journals in 2023, with regular updates to the guidelines.

### 2.11 Promotion of the guideline

After the guideline published, Jiangxi University of Chinese Medicine will popularize it through the following ways:

(1) the guideline will be suggested in 3 years at MPS conferences;
(2) the Guideline Working Group will publish the research related to guideline;
(3) Chinese and English versions of the guideline will be posted on official health websites.

And the guideline will be updated in future.

## 3 Discussion

We have developed MPS moxibustion guidelines based on the principles and standards of evidence-based medicine, in collaboration with multidisciplinary experts, which will facilitate the treatment of MPS by clinicians, as well as for teaching and educating patients.

### 3.1 Limitations

Most of the sites included in the study are in China. The application of moxibustion in other countries and regions needs further study. At the same time, the differences of moxibustion operation methods between different countries should also be considered.

### 3.2 Contributions

This study has a number of Contributions: first, to the best of our knowledge, this is the first CPG protocol to assess moxibustion therapy for patients with MPS; secondly, the results of this practice guideline protocol will be beneficial to acupuncturists and physicians to make decisions the optimal method of treating the disease, and help patients with MPS seeking optimal treatment.; finally, the results are helpful to find out the correct operation method of moxibustion for TREATING MPS and the relationship between the therapeutic effect, the time of moxibustion, and the total amount of moxibustion.

## 4 Conclusion

This guideline will be the first CPG guide for moxibustion treatment of MPS in China, which is consistent with the latest definition of the Institute of Medicine guidelines. Importantly, the guidelines will promote standards and popularize the moxibustion treatment for MPS with moxibustion, effectively improving the efficacy and safety of MPS with moxibustion.

## Acknowledgment

The authors thank the statistician group for statistical advice and randomization assistance.

## Author contributions

ZeNan Wu, Jun Xiong, and GuiXing Xu were responsible for the conception of the study and have together with QiongShan Xie planned the process analysis. Jun Xiong, ZhengYun Zuo, QiongShan Xie and ZeNan Wu formulated and composed the questionnaires. Jun Xiong, ZeNan Wu, and ZhenYun Zuo have performed the daily work with study inclusion and data collection. All authors have been involved in writing the manuscript and all authors have read and approved the final manuscript.
